# Contrast-enhanced Ultrasound in evaluating of angiogenesis and tumor staging of nasopharyngeal carcinoma in nude mice

**DOI:** 10.1371/journal.pone.0221638

**Published:** 2019-08-23

**Authors:** ShouJun Liang, Yong Gao, YaoLi Liu, ChengCheng Qiu, YanHao Chen, ShangYong Zhu

**Affiliations:** 1 Guangxi Medical University, Nanning, Guangxi, China; 2 Department of Diagnostic Ultrasound, First Affiliated Hospital of Guangxi Medical University, Nanning, Guangxi, China; University of Sheffield, UNITED KINGDOM

## Abstract

**Objective:**

To explore the use of Contrast-enhanced Ultrasound (CEUS) in evaluating angiogenesis in a xenograft nasopharyngeal carcinoma (NPC) model in nude mice and the evolution of CEUS parameters according to the growth of NPC.

**Methods:**

Nude mice were divided into three groups according to experiments conducted at various times from tumor implantation (8 mice/group; group A: 4 weeks from implantation; group B:6 weeks from implantation; group C:8 weeks from implantation). CNE-2 cells were transplanted in 24 nude mice and CEUS evaluations of the tumors were performed at 4, 6 or 8 weeks from implantation. CEUS parametric perfusion images and pathological findings were recorded. R version 3.4.4 software was used to analyze the CEUS parameters and pathological findings.

**Results:**

One-way anova analysis indicated statistically significant differences among the three groups with the parameters of peak intensity (PI) (p<0.001), area wash in (AWI) (p<0.001), area wash out (AWO) (p<0.001) and tumor volumes (p<0.001).Pearson correlation coefficient analysis indicated that microvessel density (MVD) was correlated with tumor volume (r = 0.644, p = 0.001), PI (r = 0.904, p<0.0001), AWI (r = 0.547, p = 0.008) and AWO (r = 0.744, P<0.0001). Tumor volume was correlated with MVD (r = 0.644, p = 0.001), PI (r = 0.625, p = 0.002), AWI (r = 0.528, p = 0.012) and AWO (r = 0.784, p<0.001). The percentage of necrosis in histological sections was correlated with the percentage of CEUS unperfused area (r = 0.446,p = 0.038). Spearman rank correlation coefficient analysis indicated that vascular endothelial growth factor (VEGF) was correlated with PI (r = 0.462, P = 0.032). Welch t test indicated PI, AWI and AWO parameters were significantly lower than that of kidneys (p<0.001, p = 0.009, p = 0.005).

**Conclusions:**

The CEUS parameters PI, AWI and AWO indirectly reflect the MVD and the tumor volume in our model of subcutaneous transplanted NPC in nude mice, providing precious information on angiogenesis and tumor growth. VEGF may play a role in promoting angiogenesis of NPC.

## Introduction

Nasopharyngeal carcinoma (NPC) is a common type of cancer in south of China, especially in the Guangxi province[1]. It has an unbalanced geographical distribution, and nearly 80% of new NPC cases in 2008 were in Asia and only 5% were in Europe[2]. NPC is a malignant tumor originating from the epithelium of the nasopharynx, and the lack of early stage obvious symptoms makes it hard to diagnose. The majority of NPC patients are diagnosed only at the advanced stage and the five year survival rate is 50%-60%[3].

Radiation therapy (RT) has been the primary treatment for NPC and Intensity-Modulated Radiotherapy (IMRT) is the preferred standard of care in non-metastatic NPC[4]. IMRT combined with concurrent chemotherapy or neoadjuvant-concurrent chemoradiotherapy has been proven to improve relapse-free survival and disease-free survival of NPC[5]. In recent years, RT combined with anti-angiogenic treatment has been used in clinical trials and considered a promising model for NPC treatment[6, 7].

Angiogenesis is a complex process involving endothelial cell division and migration leading to the formation of new capillaries, and is crucial to tumor growth. The new capillaries in tumors are thin-walled and leaky, exhibiting increased permeability, arteriovenous shunting, chaotic flow patterns, and fragility[8, 9]. In addition, new capillaries in tumors often present with either collapsed or blind ended sacs so that they are inefficient in carrying blood flow. Thus, tumors are often hypoxic and nutrient supply is poor, resulting in necrosis areas[10]. At the molecular level, pro-angiogenic proteins such as vascular endothelial growth factor (VEGF) can be upregulated under hypoxic conditions to promote tumor angiogenesis[11]. Microvasculature density (MVD) has been used to measure angiogenesis in tumors. Endothelial markers such as CD34 and CD31 can highlight microvessels to enable microvessel counting [12].

At present, the imaging examination of NPC mainly includes computer tomography (CT), F-fluorodeoxyglucose (FDG) positron emission tomography (PET)/CT and magnetic resonance imaging (MRI) [13, 14]. Some previous studies indicated that ultrasonography was a useful tool for diagnosing NPC and for defining the relationship between a tumor and the parapharyngeal space[15, 16], however, conventional ultrasound (US) of NPC still has some limitations. Conventional US can only provide morphological information of tumors, but it can't show tumor angiogenesis. Besides, it is not easy to diagnose whether NPC is invading bone and intracranial and paranasal sinus by conventional US[15]. Conventional Doppler imaging like color Doppler or power Doppler is not sensitive enough to study the microvasculature of tumors because blood flow is too slow[17]. Besides, Conventional Doppler imaging is limited by factors such as detection depth and angle and blood flow velocity, and therefore early diagnosis is difficult and often cannot truly reflect the internal conditions of lesions.

Contrast-enhanced ultrasound (CEUS) has been shown to be a practical method of detecting the features of tumor angiogenesis in many studies, some of which investigated the correlation between CEUS parameters and MVD or VEGF expression to assess tumor angiogenesis[18, 19]. CEUS has the ability to gather micro-vascular information in various organs, which increases the accuracy of tumor diagnosis[17, 20–24]. So far, there have been reports of CEUS in the diagnosis of lymph node metastasis of NPC [1]. However, CEUS has not until now been used in the diagnosis of primary NPC. We have no knowledge of the CEUS imaging characteristics of primary NPC angiogenesis. Thus, the present study was performed to explore the CEUS imaging blood perfusion characteristics to assess angiogenesis and tumor growth of human NPC CNE-2 cells transplanted tumors in nude mice.

## Materials and methods

### Cell culture

Human NPC CNE-2 cells were bought from Shanghai Zhong Qiao Xin Zhou Biotechnology Co., Ltd. and cultured in complete Dulbecco's Modified Eagle Medium (DMEM, Gibco, Thermo Fisher Scientific) supplemented with 10% fetal bovine serum at 37°C and 5% CO2, in a humid environment.

### Animal tumor model

A total of 24 five-week-old male BALB/c nude mice weighing 18–25g were obtained from Experimental Animal Center of Guangxi Medical University (Guangxi, China). The nude mice were fed under the Specific Pathogen Free (SPF), 24–26°C constant temperature environment. The cage equipment, bedding, drinking water and feed were disinfected and sterilized. CNE-2 cells (1 × 10^7^/mL) were suspended in 0.2 ml of phosphate buffer saline solution (PBS, 0.01 M, pH = 7.4), then subcutaneously injected into the back of each nude mouse to establish a mouse model of nasopharyngeal tumor. The nude mice were randomly divided into three groups (group A, group B and group C), 8 mice in each group. CEUS evaluations of the tumors were performed after implantation (group A: at 4th week; group B: at 6th week; group C: at 8th week). All animal experiments and procedures for animal experimental protocols were approved by the animal care and use committee of Guangxi Medical University under the guidelines of the National Institutes of Health for the care of laboratory animals.

### US examinations

For the US imaging studies, each mouse was anesthetized by inhalation of isoflurane (2% induction and 1.5% maintenance).US images of tumors were obtained on anesthetized animals using an Aplio 500 (Toshiba Medical Systems, Tokyo, Japan)US equipment associated with a 14L5 linear array transducer (5–14 MHz), mechanical index: 0.07, dynamic range: 65 dB. Settings were adjusted at the beginning and maintained constant during the experiments.

Conventional US and Color Doppler were performed on all tumors before the contrast-enhanced study. US parameters including the greatest longitudinal diameter, maximum transverse diameter and depth of tumors were measured in gray-scale imaging. Tumor volume was calculated using the formula[25]: volume = π/ 6 × length × width × depth.

US contrast agent SonoVue (Bracco, Italy) was used for CEUS imaging. It was dissolved with physiologic saline to 5 ml and was intravenously injected as a bolus (0.1 ml/20 g) via the caudal vein of nude mice[25]. Images were acquired and stored in real time. The bolus injection was performed by the same operator to minimize variations in the injection rate.

### Image analysis

Time-intensity curves (TIC) were extracted from the images and analyzed using the quantitative analysis software CHI-Q (Toshiba, Japan). A circular region of interest (ROI with a 2-mm radius) was selected inside the perfused region of the tumor. A similar ROI was drawn in the kidney of the nude mice as a reference. The percentage of unperfused area was estimated by software Image J (National Institutes of Health, USA). The following quantitative CEUS parameters were extracted from the TIC calculated in the ROI: (1) peak intensity (PI), (2) time to peak (TTP), (3) mean transit time (MTT), (4) area wash in (AWI), (5) area wash out (AWO). The parameters of the tumor were divided the parameters by the ones obtained in the kidney to normalize (PI tumor/PI kidney, TTP tumor/TTP kidney, MTT tumor/MTT kidney, AWI tumor/AWI kidney, AWO tumor/AWO kidney). The images were analyzed and agreed upon by two experienced sonographers.

### Immunohistochemical analysis

After CEUS imaging measurement for each group, nude mice were euthanized by CO2 overdose, the tumors were quickly extracted and fixed in 10% formalin. The tumor tissues were dehydrated and paraffin embedded, sliced, and were hematoxylin and eosin (H&E) stained or used for immunohistochemical staining. A Rabbit anti-mouse CD34 antibody (Abcam, UK), and an anti-VEGF antibody (Abcam, UK) were the primary antibodies used in immunohistochemistry. Polymer helper and polyperoxidase-anti-rabbit IgG were from Zhongshan Goldenbridge Biology, Beijing, China and diaminobenzidine (DAB) was used for color development. Immunohistochemical staining was performed according to the standard procedures.

CD34 was the staining used for MVD estimation. Tumor MVD was measured according to an established method by Weidner et al[26]. Areas of tumor were examined by light microscopy to locate brown stained microvessels. Firstly, three areas of the highest neovascularization (hot spots) were found by scanning the tumor sections at low power (100 X total magnification). Secondly, the numbers of individual brown-stained cells were counted at high power (200 X total magnification) for MVD, regardless of whether the microvessel cavity was formed or not. Finally, the mean value of the microvessels in the three hot spots was the result of MVD for the slice.

Concerning the method of evaluating VEGF expression, three areas of cytoplasmic and membrane brown stained positive cell dense were found at low power (100X), then transferred to high power (200X). Sections of VEGF stained were scored semi-quantitatively for immunoreaction as follows: 0: 0% of immunoreactive cells; 1: <25% of immunoreactive cells; 2: 25–50% of immunoreactive cells; and 3: >50% of immunoreactive cells. Also, intensity of staining was scored semi-qualitatively as follows: 0: negative; 1: weak; 2: intermediate; and 3: strong. The score of each area was the sum of both parameters, the final score was defined as the mean value of the three areas. Immunohistochemical evaluation was performed blindly by two independent observers.

### Statistical analysis

All the data were analyzed by R version 3.4.4 software. One-way anova was used to analyze the significant differences among the three groups with the parameters and the tumor volume. Pearson correlation coefficient was used to analyze the correlation between CEUS parameters and MVD and tumor volume; also the correlation between the percentage of necrosis in histology and the percentage of unperfused area established by CEUS. Spearman rank correlation coefficient was used to analyze the correlation between VEGF and CEUS parameters and MVD. Welch t test was used to analyze the significant differences of CEUS parameters between tumors and kidneys of mice. Values are expressed as mean ± standard deviation (SD). P < 0.05 was considered statistically significant.

## Results

### Pathological features of NPC

**Fig 1:**
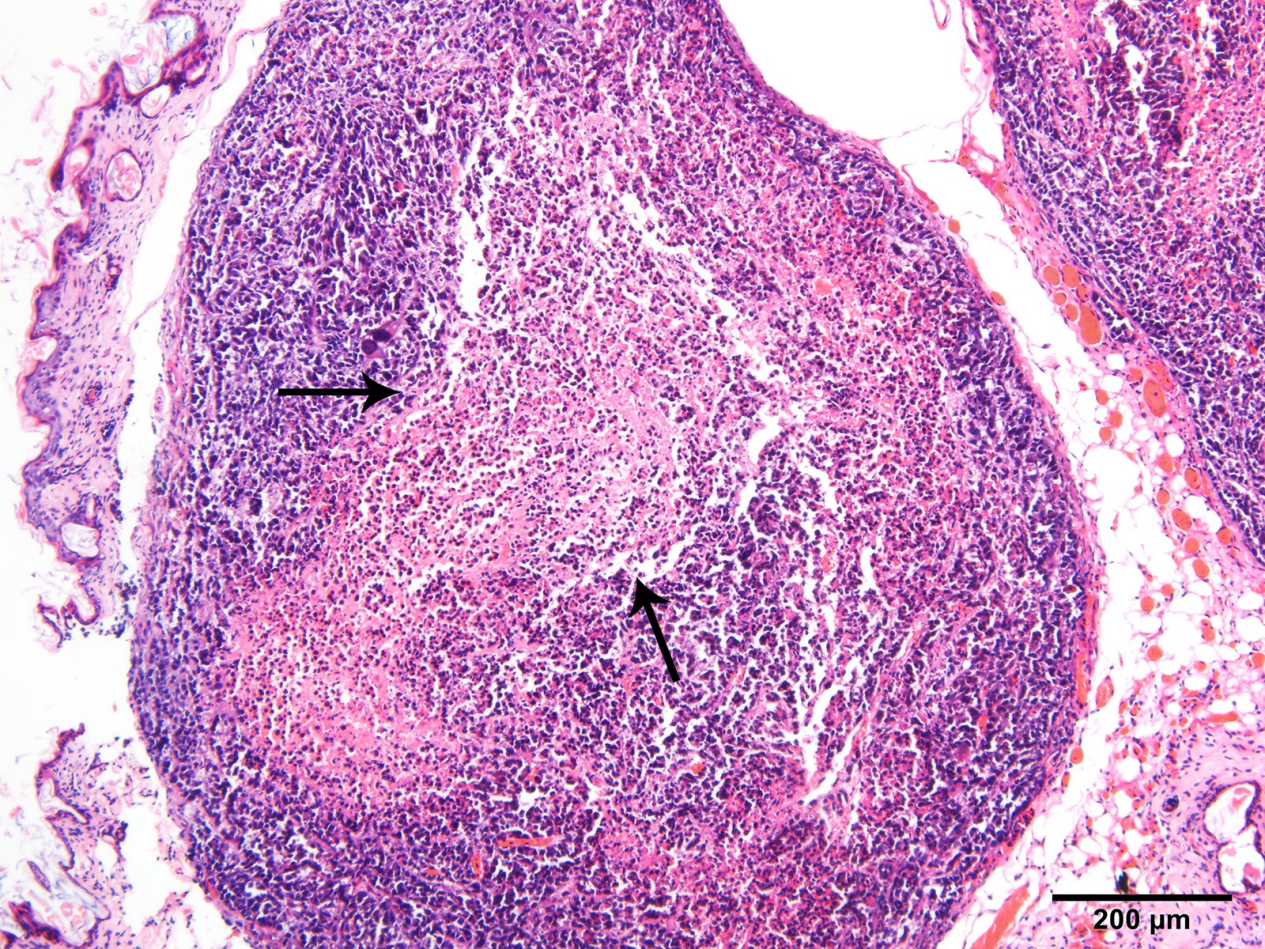
HE staining of NPC (100X). Black arrows designate necrotic areas in the center of the tumor. The periphery of the tumor are nasopharyngeal carcinoma cells.

**Fig 2:**
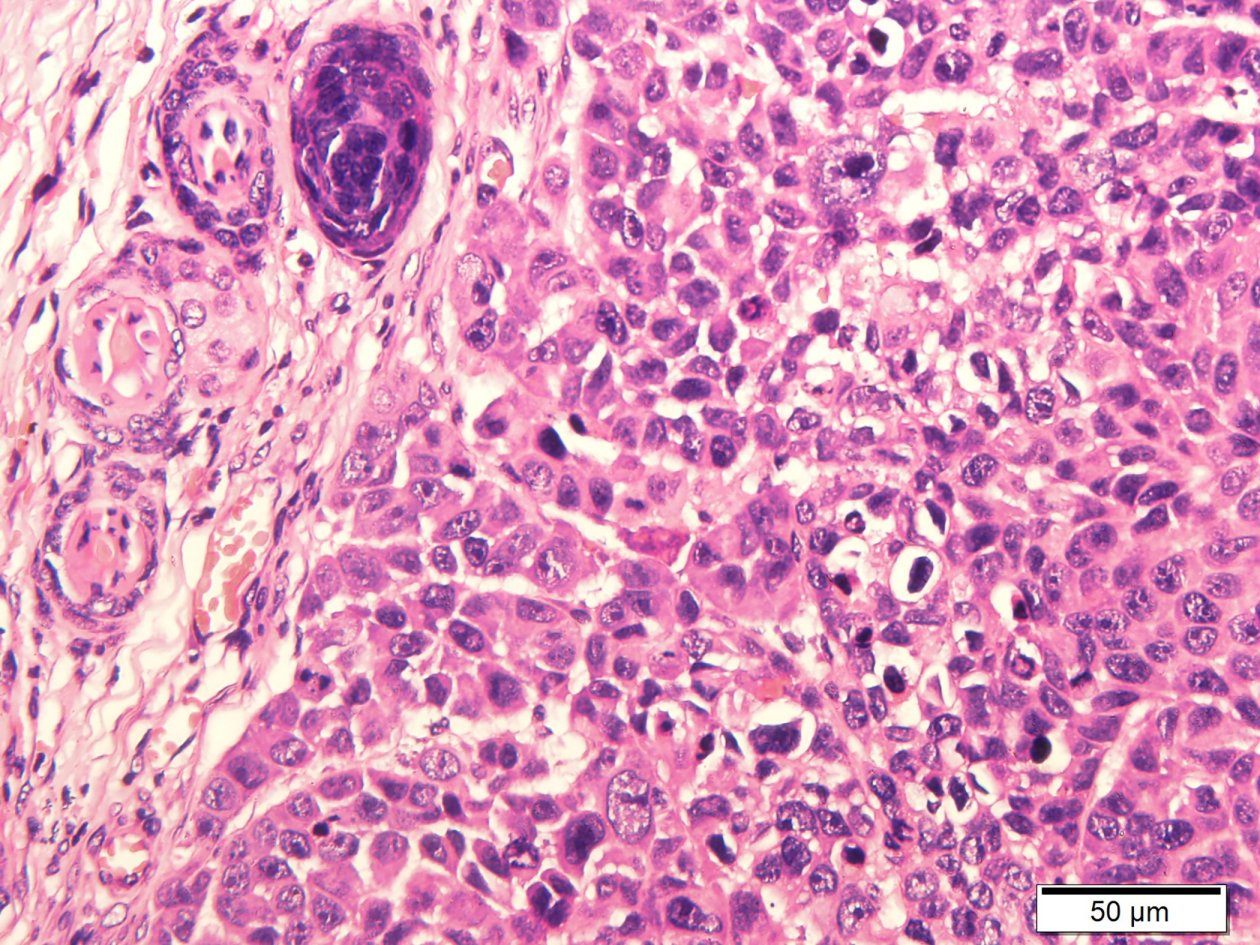
HE staining of NPC (400X).

**Fig 3:**
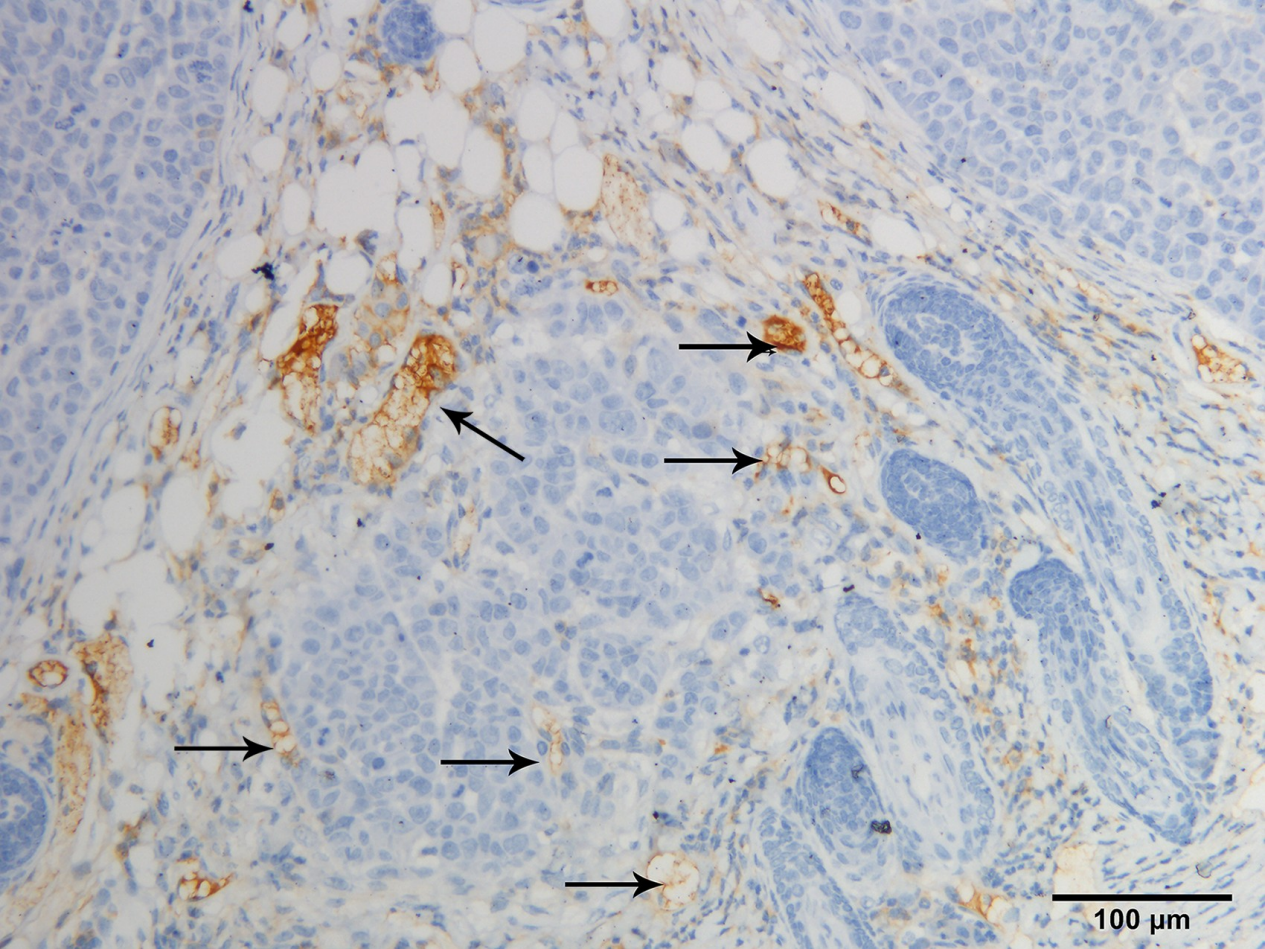
All microvessels were strongly labeled by CD34 (200X). Black arrows designate endothelial cells.

**Fig 4:**
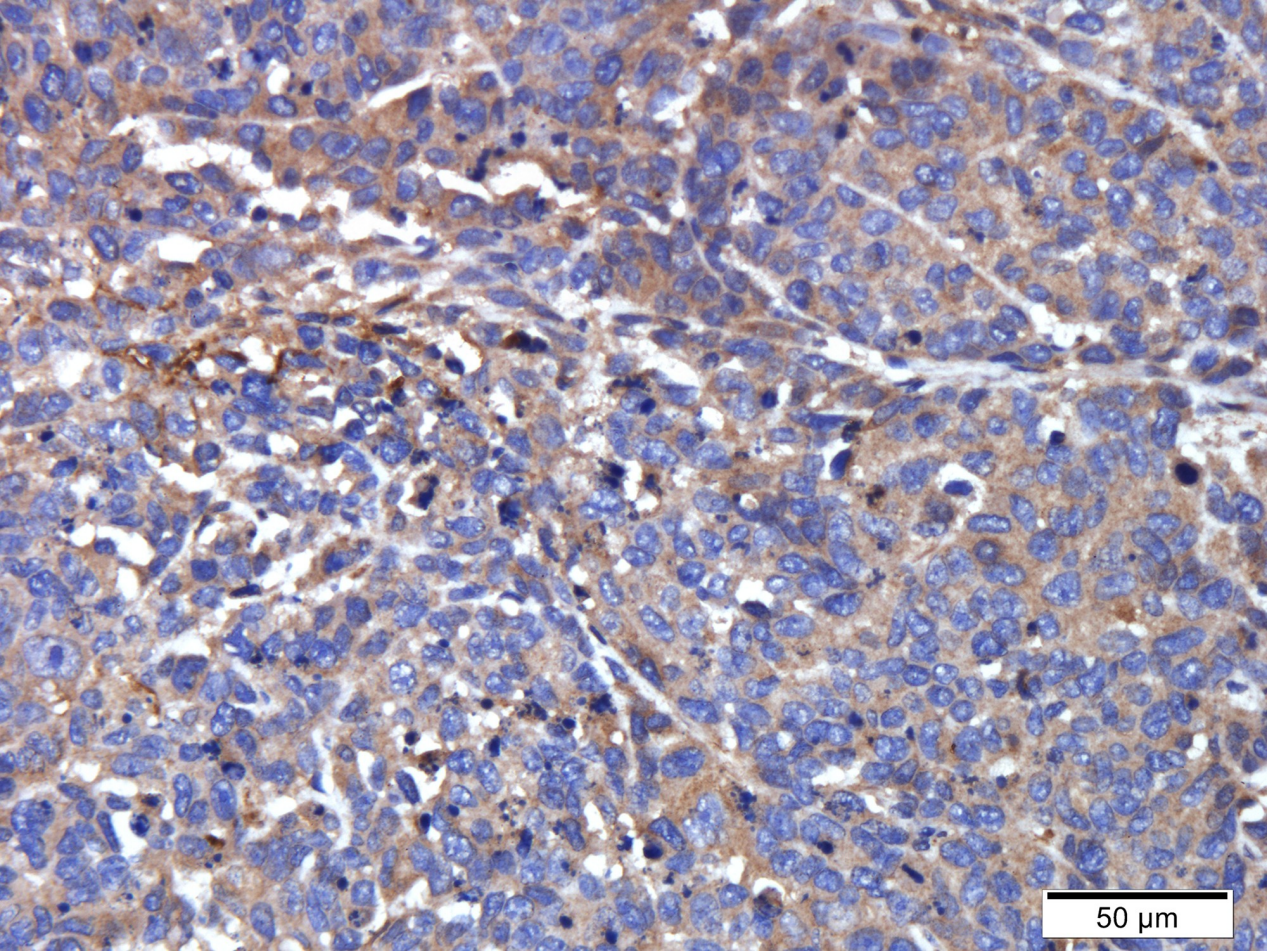
Immunohistochemical staining of the tumor tissues with the anti-VEGF antibody (400X).

Tumor specimens were pale and soft. HE staining showed necrosis in the center of some tumors (Fig 1). The central cells of the tumor had obvious heteromorphism, disordered arrangement, large deep staining of nuclei and common nuclear fission images (Fig 2). CD34 staining showed that vascular endothelial cells were brownish yellow stained, which were most frequent at the margins of the tumor or invasive periphery (Fig 3). VEGF staining showed brown staining of cytoplasm or cell membranes (Fig 4). Comparing with necrosis seen on H&E, CD34 staining and VEGF staining positive expressions were mostly distributed in non-necrotic areas of the tumor. This indicated that the effective blood supply of neovascularized tumor was mainly distributed at the periphery of the tumor. There was no correlation between MVD and VEGF (r = 0.327, p = 0.138).

### Conventional US detection of NPC

**Fig 5:**
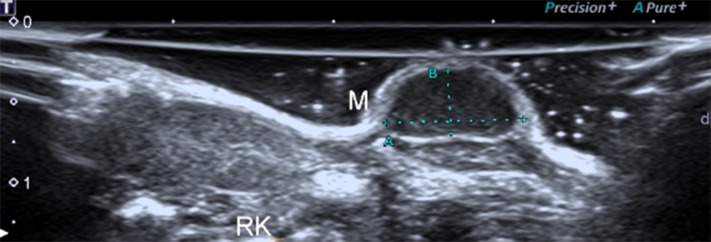
B-mode US detection of NPC. The tumor was indicated with the letter “M”.

**Fig 6:**
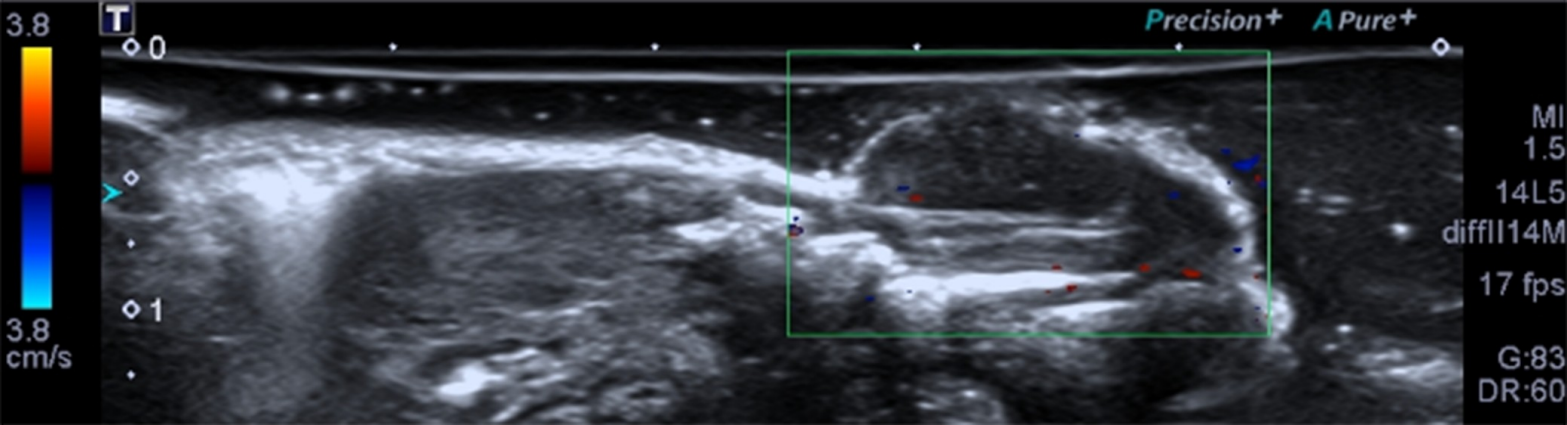
Color Doppler detection of NPC.

The tumors presented round or oval shapes with clear boundaries around a hypo-echoic mass. The internal echo pattern was inhomogeneous (Fig 5). Color Doppler flow imaging (CDFI) showed that the blood flow of NPC lesions was not abundant, there was only sparse blood flow appearing around or inside the lesions. (Fig 6).

### CEUS features

Two mice in group C could not be injected with SonoVue through the caudal vein, and therefore a total of 22 cases of successful CEUS data were recorded.

**Fig 7:**
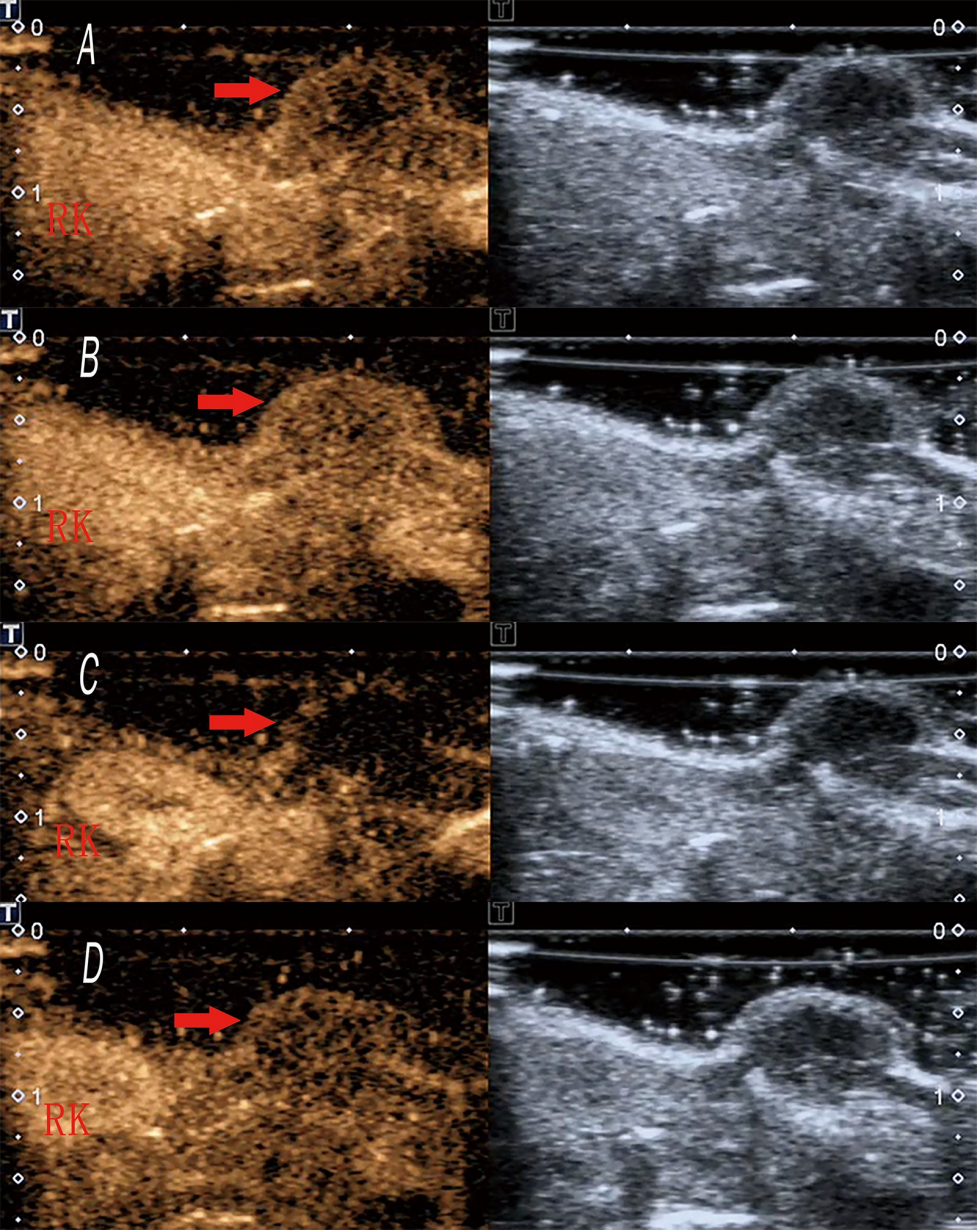
CEUS features of NPC. (A) Rapid peripheral annular enhancement in the early stage of NPC in nude mice (NPC is indicated with an arrow). (B) The interior portion of the NPC periphery showed perfusion with heterogeneous enhancement (NPC is indicated with an arrow). (C) The beginning of the contrast enhancement came later in tumors than in the adjacent kidney (indicated with the letter “RK”)of nude mice(NPC is indicated with an arrow). (D) The contrast agent began to recede in tumors earlier than that in the kidney (indicated with the letter “RK”) of the nude mice (NPC is indicated with an arrow).

CEUS showed rapid peripheral annular enhancement in the early stage of subcutaneous transplantation NPC tumor in nude mice (Fig 7A). The peripheral portion of the tumor showed perfusion with heterogeneous enhancement firstly, then the contrast agent gradually perfused into the interior of the tumor and the degree of enhancement was weaker than that of the peripheral part of the tumor (Fig 7B). However, the beginning of the contrast enhancement came later in tumors than in the adjacent kidney (Fig 7C). In addition, the contrast agent began to recede in tumors earlier than that in the kidney (Fig 7D).

### Parametric perfusion images evaluation

**Fig 8:**
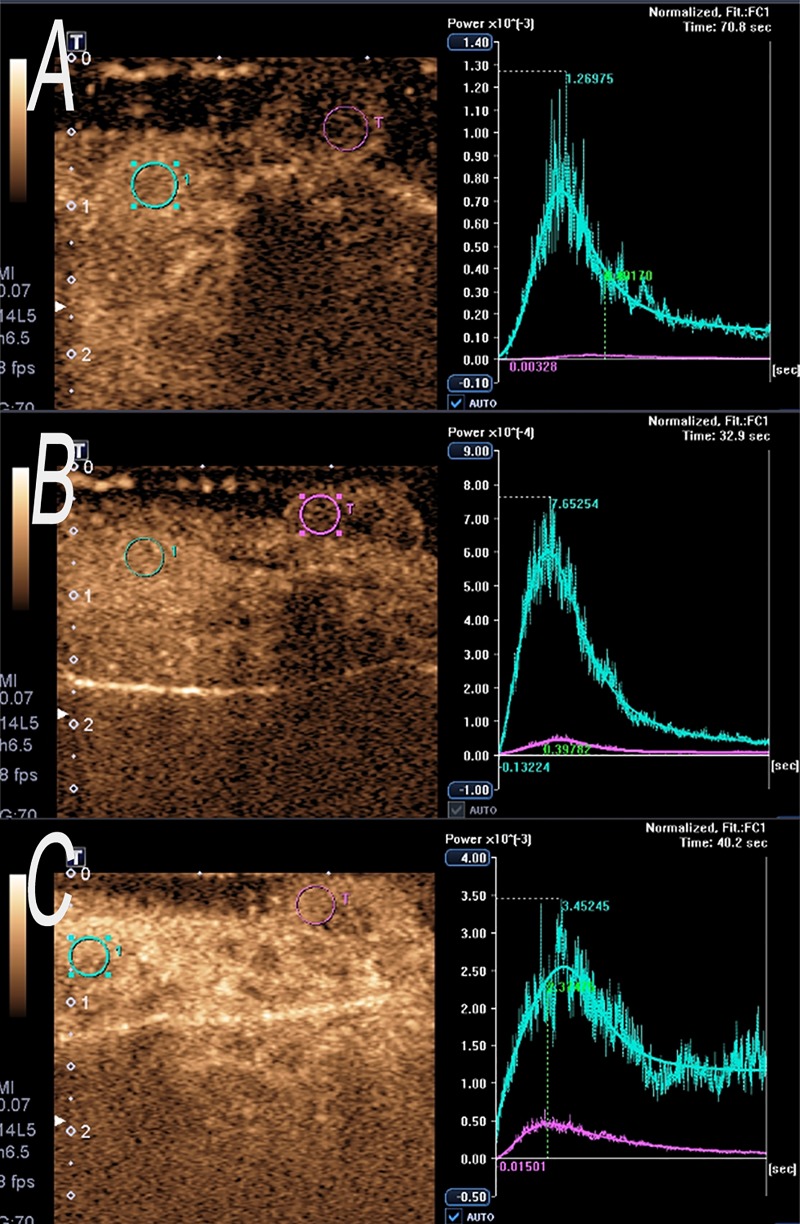
Time-intensity curve of CEUS. (A) One example of the TICs extracted from the tumor and kidney ROIs for the group A. (B) One example of the TICs extracted from the tumor and kidney ROIs for the group B.(C) One example of the TICs extracted from the tumor and kidney ROIs for the group C.

The value of the CEUS parameters was extracted from the TIC for the three groups (Fig 8A–8C).The mean CEUS perfusion parameters of NPC in the three groups were summarized in Table 1. We used a logarithmic transformation to make the data satisfy the normal requirement. The transformed data was used in an Anova test. PI was significantly higher for group C (15.62±5.68) than for group A (2.29±0.96) (P < 0.001) and group B (9.14±1.68) (p = 0.003). PI for group A was significantly lower than for group B (p<0.001).

**Table 1:** The CEUS perfusion parameters and immunohistochemical analysis results of NPC in the Three groups.

	Group A (mean±SD)	Group B (mean±SD)	Group C (mean±SD)
**PI (10E-5AU)**	2±1	9±2	16±6
**TTP (S)**	29±6	43±20	41±23
**MTT (s)**	50±13	71±39	93±53
**AWI (10E-5AU·s)**	42±21	245±137	337±146
**AWO (10E-5AU·s)**	104±46	432±275	964±181
**MVD**	7±1	10±2	11±2
**VEGF**	2±1	3±2	4±1

AWI was significantly higher for group C (336.75±145.63) than for group A (41.80±21.24) (P < 0.001). AWI for group B (244.95±136.68) was significantly higher than for group A (p = 0.005). There was no significant difference assigned to the AWI parameter between group B and group C (p = 0.308).

AWO was significantly higher for group C (963.78±181.49) than for group A (103.73±46.34) (P < 0.001) and group B (431.84±275.21) (p<0.001). AWO for group B was significantly higher than for group A(p = 0.008).

### Tumor volume evaluation

**Fig 9:**
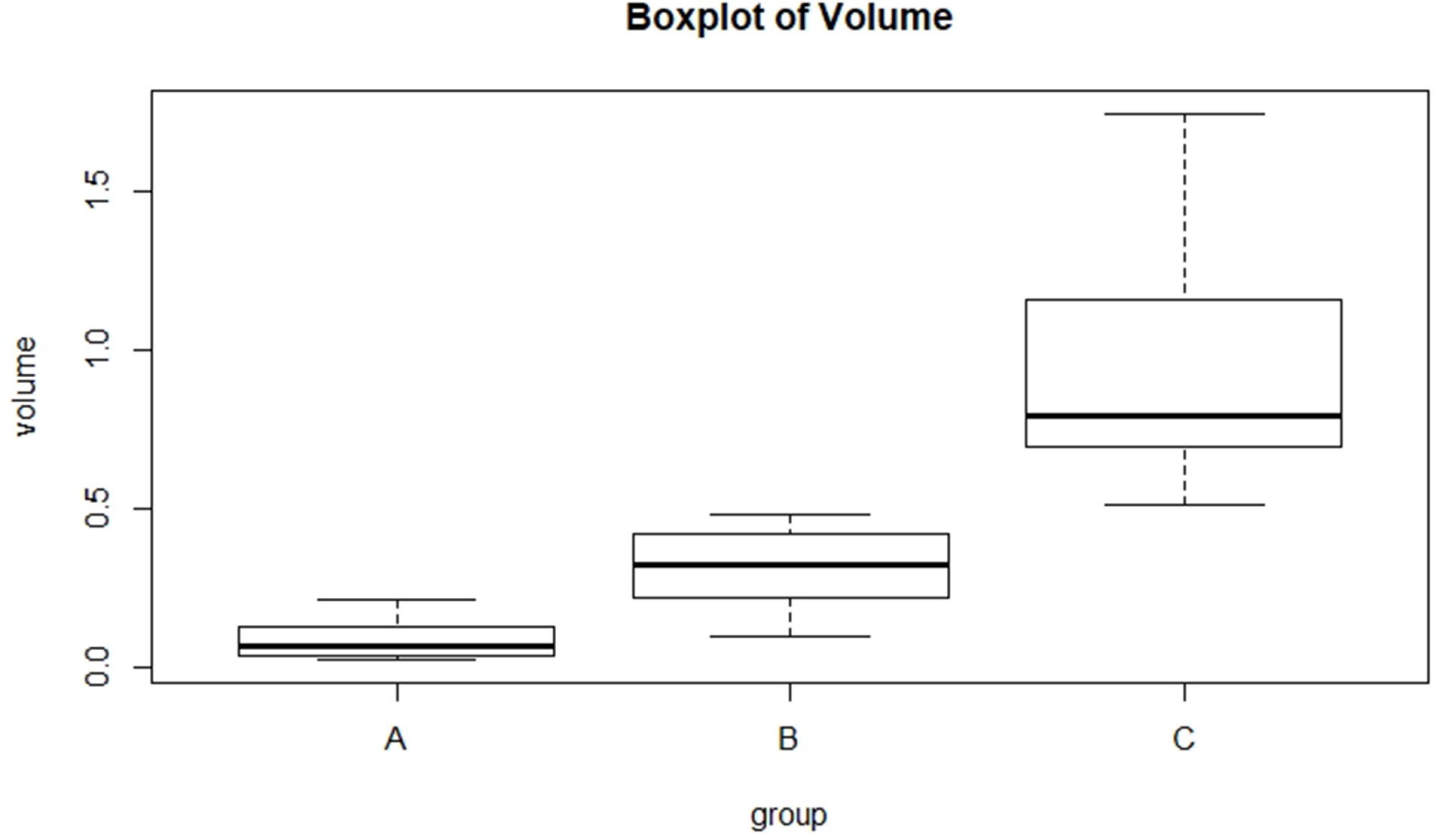
Comparion of tumor volume among three groups.

**Fig 10:**
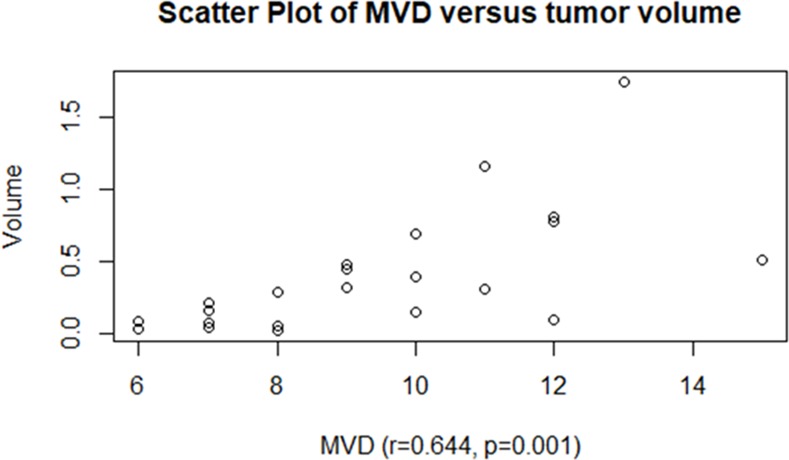
Correlations between MVD and the tumor volume.

The tumor volume was significantly larger for group C (8 weeks, 0.95±0.44 cm3) than for group A (4 weeks, 0.09±0.07 cm3) (p<0.001) and group B (6 weeks, 0.31±0.13 cm3) (p<0.001). There was no significant difference between group A and group B (p = 0.190)(Fig 9). The tumor volume was positively correlated with MVD (r = 0.644, p = 0.001)(Fig 10).

### Correlation of parametric perfusion images and immunohistochemical finding

Pearson’s *r* was used to evaluate the relationship between the percentage of necrosis in histology and the percentage of CEUS unperfused area.

**Fig 11:**
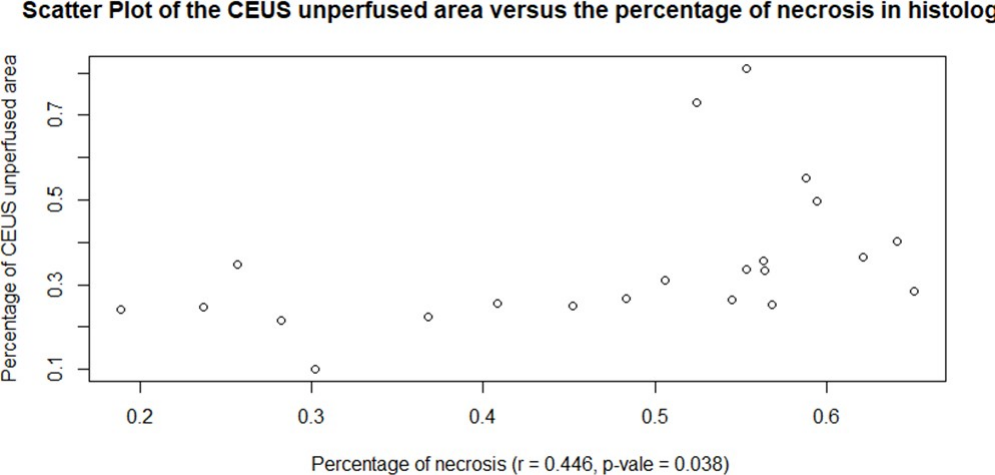
Correlations between the CEUS unperfused area and the percentage of necrosis by histology.

We selected the CEUS image at the moment when the tumor enhancement reached the PI in all the mice to estimate the percentage of unperfused area in the tumor. We compared the size of unperfused area (35% ±17%) measured on CEUS images with the necrosis area (47%±14%) estimated on H&E sections; the percentage of necrosis in histology was correlated with the percentage of CEUS unperfused area (r = 0.446,p = 0.038)(Fig 11).

We made the immunohistochemistry analysis in a similar region to the ROI where we quantified CEUS parameters. The relationship between MVD and CEUS parameters was measured by Person’s *r*, since MVD was a continuous variable. The relationship between VEGF and CEUS parameters was measured by Spearman’s *r*, since VEFG was ordinal variable. The parameter of PI was positively correlated with MVD (r = 0.904, p<0.0001) and VEGF (r = 0.462, P = 0.032); AWI was positively correlated with MVD (r = 0.547, p = 0.008); AWO was positively correlated with MVD (r = 0.744, P<0.0001) (Tables 2 and 3).

**Table 2:** Pearson correlation analysis of CEUS perfusion parameters with MVD of NPC.

MVD	PI	TTP	MTT	AWI	AWO
**Correlation coefficient**	0.904	0.145	0.239	0.547	0.744
**P-value (2-tailed)**	<0.0001	0.520	0.284	0.008	<0.0001

**Table 3:** Spearman correlation analysis of CEUS perfusion parameters with VEGF.

VEGF	PI	TTP	MTT	AWI	AWO
**Spearman rho**	0.426	0.138	-0.185	0.390	0.280
**P- value**	0.032	0.539	0.410	0.074	0.207

### Correlation of parametric perfusion images and the tumor volume

Person’s *r* was used to evaluate the relationship between the tumor volume and CEUS parameters, since the tumor volume was continuous variable. The tumor volume was positively correlated with PI (r = 0.625, p = 0.002), AWI (r = 0.528, p = 0.012) and AWO (r = 0.784, p<0.001).

### Comparisons of CEUS perfusion parameters in NPC and kidney

The results of homogeneous variance test and normality test showed that all the variables were non-homogeneous and drawn from non-normal distribution. Therefore, Welch’s test was applied to verify the mean difference of NPC CEUS parameters and Kidney CEUS parameters, since the assumptions of Student’s T test was not satisfied.

PI, AWI and AWO was significantly lower for NPC than for kidney (p<0.001, p = 0.009, p = 0.005) (Table 4).

**Table 4:** Welch t test of comparisons of CEUS perfusion parameters in NPC and kidney.

	NPC(mean±SD)	Kidney(mean±SD)	P -value
**PI (10E-5AU)**	8±6	163±185	<0.001
**TTP (S)**	37±18	34±22	0.618
**MTT (s)**	69±39	93±59	0.120
**AWI (10E-5AU·s)**	196±164	2466±3699	0.009
**AWO(10E-5AU·s)**	458±394	9459±13309	0.005

## Discussion

In our study, we explore the CEUS in evaluating angiogenesis and tumor growth in a model of subcutaneous transplanted NPC in nude mice. We found that with the growth of the NPC, some CEUS parameters increased similarly and were correlated with the immunohistochemical results of the NPC.

### CEUS features of NPC transplantation tumor

NPC tumors showed rapid highly enhancement in peripheral tissues on CEUS images. However, the center of some tumors showed low to no enhancement. This can be explained by the fact that new capillaries in NPC are mostly present in the periphery of the tumor, while the center of the tumor is poorly supplied. The human NPC CNE-2 cells are prone to necrosis in the core of the tumor in nude mice in subcutaneous transplanted models. The enhancement characteristic of NPC was just consistent with the pathological observation of NPC tissues. Similar enhancement characteristics were reported in a lung peripheral VX2 tumor model in the rabbit.[27]. In comparison to the kinetics seen in the adjacent kidney, the contrast agents arrived later and receded earlier in the NPC. In addition, the intensity of the contrast enhancement in NPC was weaker than that in the kidney of the nude mice.

### CEUS evaluation of angiogenesis and tumor perfusion in NPC

Tumor angiogenesis is an essential step in tumor growth and metastasis[28], and several studies have shown that alteration in MVD of NPC is associated with tumor growth [29, 30].

In the present study, CEUS was performed in mice of three groups (A, B and C) at 4, 6 and 8 weeks after tumor transplantation, respectively. PI, AWI and AWO were significantly higher for 6 week tumors than for 4 week tumors. PI and AWO were significantly higher for 8 week tumors than for 6 week tumors and there was no significant difference assigned to the AWI parameter between 8 week and 6 week tumors. Parameters PI, AWI and AWO are related to blood volume and they can measure the perfusion of the micro-vasculature of a tumor [31]. In our study, PI, AWI and AWO gradually increased over time along with MVD, reflecting an increase in blood volume in the tumor resulting from neovascularization. This suggests that subcutaneous transplantation of NPC in nude mice is a vascular dependent tumor and we can infer on the density of micro-vessels in NPC using CEUS.

Since CEUS parameters could not be extracted from the tumor core due to low perfusion, a comparison between core tumor and peripheral tumor was not possible for statistical analysis. However, we compared the size of unperfused area measured on CEUS images with the necrosis area estimated on H&E slices, and the percentage of necrosis in histology was correlated with the percentage of CEUS unperfused area. In other words, CEUS was able to detect necrotic areas in the NPC. Although we made the immunohistochemistry analysis within a similar region to the ROI where we quantified CEUS parameters, there was still inherent limitation of comparison between in-vitro immunohistochemistry with 2D in-vivo imaging. In order to evaluate the tumor perfusion, the US probe is fixed on the plane with the largest tumor dimension for CEUS examination. CEUS could only display the enhanced image of a certain section of the tumor, but could not completely display the three-dimensional structure of tumor trophoblastic vessels. So, CEUS imaging might not be completely consistent with the pathological result of the slice.

To date, there have been many studies showing that anti-angiogenic therapy might be a good approach for the treatment of NPC[32–36]. These studies found that central tumor necrosis and consequential shrinkage of tumor volume occurred and tumor MVD decreased with anti-angiogenic therapy which remarkably suppressed the growth of NPC. Therefore, the combination of RT and anti-angiogenic therapy might enhance the efficacy of the antitumor effect on NPC. Our study demonstrates that CEUS can indirectly indicate angiogenesis in NPC. By comparison of parametric images of PI, AWI and AWO before and after anti-angiogenic therapy, we speculate that CEUS can play an important role in evaluating the efficacy of anti-angiogenic therapy of NPC.

### Evolution of CEUS parameters according to tumor growth

With the growth of the tumor, the oxygen and nutrients supply of the tumor mainly comes from the neovascularization. The increased neo-vascular supply gradually increases tumor volume and can facilitate tumor metastasis[11]. In our study, NPC tumor volume was significantly larger for group C (8 weeks) than for group A (4 weeks) and group B (6 weeks), indicating that the tumor volume was gradually increasing. Meanwhile, the mean perfusion showed a similar increase. Some studies investigated the influence of angiogenesis on tumor growth by comparing the tumor microvasculature change after anti-angiogenic treatment, using CEUS imaging[31, 37]. In the present study, we also found a positive correlation between tumor volume and MVD in NPC. These results mean that we can evaluate the different growth period and the blood supply of NPC through the changes of CEUS parameters. What's more, CEUS can help to define tumor boundaries more precisely, so it will be another great method to evaluate tumor metastasis and the changes of tumor morphology of NPC after RT and chemotherapy.

### The correlation between tumor perfusion and VEGF

VEGF has been revealed to be one of the critical factors for tumor growth and angiogenesis which may lead to metastasis[28]. As far as we know, there have been some studies indicating that CEUS parameters were correlated with the expression of VEGF in patients with malignant tumors[38, 39]. In addition, some studies found that the MVD was positively correlated with VEGF in the NPC[40, 41].

In our study, although we did not find any correlation between VEGF and MVD in NPC, CD34 staining and VEGF positive staining were both mostly distributed at the margins of the NPC or at invasive periphery, which matched the CEUS perfusion characteristic of the NPC. Meanwhile, VEGF and MVD were positively correlated with PI. As a result, PI can reflect angiogenesis in NPC and VEGF may play a role in promoting angiogenesis of NPC.

## Conclusion

In conclusion, our study demonstrates that the CEUS is able to detect necrotic areas in NPC. The CEUS parameters PI, AWI and AWO indirectly reflect the MVD and the tumor volume in our model of NPC subcutaneous transplanted nude mice, providing precious information on angiogenesis and tumor growth. VEGF may play a role in promoting angiogenesis of NPC. CEUS can become an effective tool for the clinical characterization of NPC in the future.

## Supporting information

S1 Fig HE staining of nasopharyngeal carcinoma (100X). Black arrows designate necrotic areas in the center of the tumor.
(TIF)

S2 Fig HE staining of nasopharyngeal carcinoma (100X). Black arrows designate necrotic areas in the center of the tumor.
(TIF)

S1 File The Tab of Animal Experimental Ethical Inspection. (PDF)
