# Supplementary figures and images for: Contrast-enhanced Ultrasound in evaluating of angiogenesis and tumor staging of nasopharyngeal carcinoma in nude mice
Source: PLoS One. 2019 Aug 23;14(8):e0221638. doi: 10.1371/journal.pone.0221638 (PMC6707564; doi:10.1371/journal.pone.0221638)

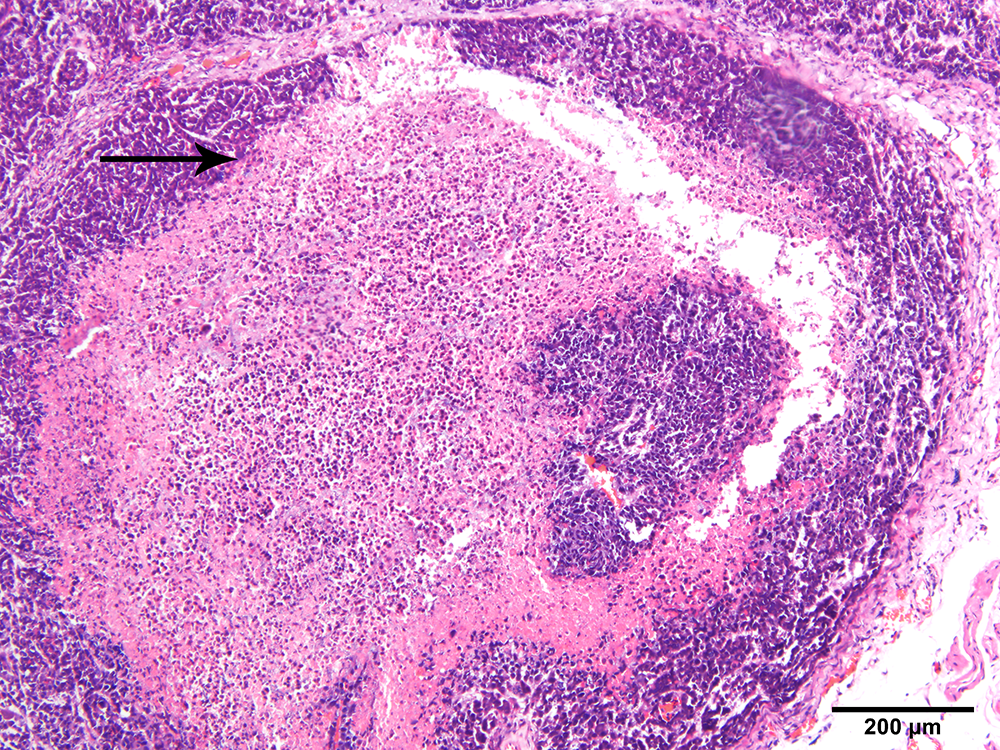

Supplement: S1 Fig — Black arrows designate necrotic areas in the center of the tumor. (TIF) [file pone.0221638.s001.tif]

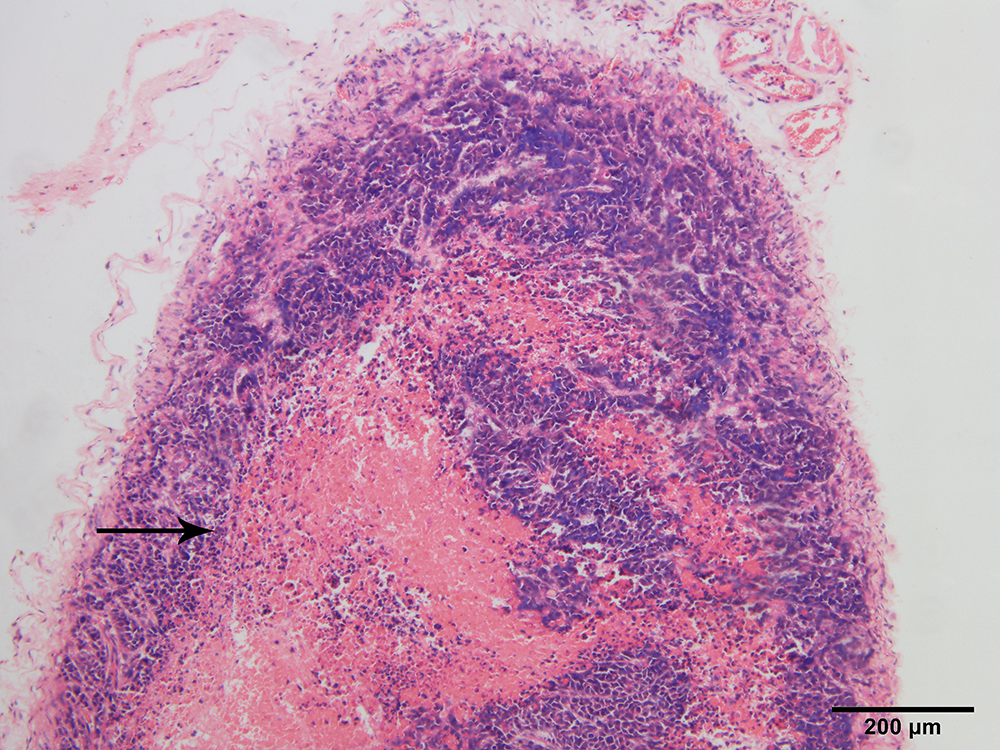

Supplement: S2 Fig — Black arrows designate necrotic areas in the center of the tumor. (TIF) [file pone.0221638.s002.tif]
