# Supplementary material for: Contrast-enhanced Ultrasound in evaluating of angiogenesis and tumor staging of nasopharyngeal carcinoma in nude mice
Source: PLoS One. 2019 Aug 23;14(8):e0221638. doi: 10.1371/journal.pone.0221638 (PMC6707564; doi:10.1371/journal.pone.0221638)
Supplement: S1 File — (PDF) [file pone.0221638.s003.pdf]

The Tab of Animal Experimental Ethical Inspection    No: 201708008

<table>
<tr><td rowspan="9">Concerned information written by applicant</td><td colspan="3">Applicant: Shoujun Liang</td></tr>
<tr><td colspan="3">Education of applicant: Master Degree    Professional title: Resident Doctor</td></tr>
<tr><td colspan="3">Study title: Animal and human experiment study of contrast-enhanced ultrasound in the diagnosis of nasopharyngeal carcinoma</td></tr>
<tr><td colspan="3">Aim of experiment: To explore the diagnostic and differential diagnostic value of Contrast-enhanced Ultrasound (CEUS) for nasopharyngeal carcinoma (NPC) transplantation tumor and explore the efficiency of CEUS in examination of changes in NPC before and after radiotherapy in nude mice models.</td></tr>
<tr><td colspan="3">Fund sources: The National Natural Science Foundation of China(grant number: 81560288)</td></tr>
<tr><td rowspan="4"></td><td colspan="2">Source of animal: 1、Experimental Animal Center of Guangxi Medical University<br>2、Hunan SJA Laboratory Animal Co., Ltd</td></tr>
<tr><td colspan="2">Species or strain: BALB/c-nu    Grade: SPF    Specifications: 4 weeks</td></tr>
<tr><td>Number: 100 (♀50 ; ♂ 50)</td><td>Application date: August 2017</td></tr>
<tr><td>Entering date: August 2017</td><td>Ending date: December 2019</td></tr>
<tr><td></td><td colspan="3">Outline of experiments; experimental methods; observational index:<br>1. The nude mice are randomly divided into NPC group、Inflammatory group、Radiotherapy group and Hemangioma group.<br>2. Establish mice model of xenografts and use an Aplio 500 ultrasound equipment to obtain CEUS images of NPC、inflammatory mass and hemangioma.<br>3. Analyze the CEUS quantitative parameters: (1) peak intensity (PI), (2) time to peak (TTP), (3) mean transit time (MTT), (4) area wash in (AWI), (5) area wash out (AWO).<br><br>Human endpoint or experimental terminative indicator:<br>When the length, width and thickness of the tumor approach 2 cm.<br><br>Executing animal method:<br>Nude mice were euthanized by CO2 overdose.<br><br>Major measure for 3Rs:<br>1、Strengthen nutrition for nude mice, improve experimental efficiency and shorten experimental time to improve the tolerance of nude mice and prolong life.<br>2、Establish models on both sides of the back of nude mice to improve the success rate of experiments.<br>3、Each mouse was anesthetized by inhalation of isoflurane (2% induction and 1.5% maintenance) through a Gas filter canister R510-31 (RWD, China).</td></tr>
<tr><td></td><td colspan="3">Signature of applicant: Shoujun Liang [signature]    Telephone: 13197616366</td></tr>
</table>

| | |
|---|---|
| Results of inspection | Study director: Shangyong Zhu Agree ☑ Disagree ☐ Signature |
| | Opinion from laboratory animal facility:<br>Agree) ☑ Disagree ☐<br>Signature |
| | The Animal Care & Welfare Committee:<br>Agree) ☑ Disagree) ☐<br>Stamp 广西医科大学实验动物伦理委员会 |
| Supplement:<br>First trial ☑ reexamine No. | |

Notes : Animal ethics review follows the *Guiding Opinions on the Treatment of Laboratory Animals* issued by the Ministry of Science and Technology of the People's Republic of China and the *Laboratory Animal-Guideline for Ethical Review of Animal Welfare* issued by the National Standard GB/T35892-2018 of the People's Republic of China. If there are any uncertainties, please refer to these two documents or consult The Animal Care & Welfare Committee of Guangxi Medical University.
